# An education initiative modifies opinions of hemodialysis nurses towards home dialysis

**DOI:** 10.1186/s40697-015-0051-z

**Published:** 2015-04-28

**Authors:** Matthew Phillips, Colleen Wile, Carolyn Bartol, Cynthia Stockman, Minakshi Dhir, Steven D Soroka, Jay Hingwala, Joanne M Bargman, Christopher T Chan, Karthik K Tennankore

**Affiliations:** Capital District Health Authority/QEII Renal Program, Halifax, Nova Scotia Canada; Division of Nephrology, Dalhousie University, Halifax, Nova Scotia B3H 1V8 Canada; Health Sciences Center/Manitoba Renal Program, Winnipeg, Manitoba Canada; Division of Nephrology, University Health Network, University of Toronto, Toronto, Ontario Canada

**Keywords:** Attitudes, Continuing nursing education, Dialysis nurse, Home hemodialysis, Perceptions, Peritoneal dialysis

## Abstract

**Background:**

It has been shown that in-center hemodialysis (HD) nurses prefer in-center HD for patients with certain characteristics; however it is not known if their opinions can be changed.

**Objective:**

To determine if an education initiative modified the perceptions of in-center HD nurses towards home dialysis.

**Design:**

Cross-sectional survey of in-center HD nurses before and after a three hour continuing nursing education (CNE) initiative. Content of the CNE initiative included a didactic review of benefits of home dialysis, common misconceptions about patient eligibility, cost comparisons of different modalities and a home dialysis patient testimonial video.

**Setting:**

All in-center HD nurses (including those working in satellite dialysis units) affiliated with a single academic institution

**Measurements:**

Survey themes included perceived barriers to home dialysis, preferred modality (home versus in-center HD), ideal modality distribution in the local program, awareness of home dialysis and patient education about home modalities.

**Methods:**

Paired comparisons of responses before and after the CNE initiative.

**Results:**

Of the 115 in-center HD nurses, 100 registered for the CNE initiative and 89 completed pre and post surveys (89% response rate). At baseline, in-center HD nurses perceived that impaired cognition, poor motor strength and poor visual acuity were barriers to peritoneal dialysis and home HD. In-center HD was preferred for availability of multidisciplinary care and medical personnel in case of catastrophic events. After the initiative, perceptions were more in favor of home dialysis for all patient characteristics, and most patient/system factors. Home dialysis was perceived to be underutilized both at baseline and after the initiative. Finally, in-center HD nurses were more aware of home dialysis, felt better informed about its benefits and were more comfortable teaching in-center HD patients about home modalities after the CNE session.

**Limitations:**

Single-center study

**Conclusions:**

CNE initiatives can modify the opinions of in-center HD nurses towards home modalities and should complement the multitude of strategies aimed at promoting home dialysis.

**Electronic supplementary material:**

The online version of this article (doi:10.1186/s40697-015-0051-z) contains supplementary material, which is available to authorized users.

## Abrégé

**Contexte:**

Le personnel infirmier s’occupant de patients qui reçoivent leur traitement d’hémodialyse (HD) en centre hospitalier préfère cette modalité thérapeutique pour certains types de patients; nous ne savons toutefois pas si cette opinion peut être modifiée.

**Objectifs:**

Déterminer si une intervention d’éducation a modifié la perception de l’hémodialyse à domicile par le personnel infirmier s’occupant des patients en HD hospitalière.

**Type d’étude:**

Étude transversale auprès du personnel infirmier en HD hospitalière, avant et après la réalisation d’une intervention d’éducation en soins infirmiers de trois heures. Le contenu de la formation comprenait un examen didactique des avantages de l’hémodialyse à domicile, des idées fausses sur l’admissibilité des patients, une comparaison des coûts des différentes modalités, de même qu’une vidéo présentant des témoignages de patients en hémodialyse à domicile.

**Contexte:**

Tout le personnel infirmier en HD hospitalière (qui comprend celui des services satellites de dialyse) affilié à un établissement d’enseignement unique.

**Méthodes:**

Les thèmes du sondage comprenaient les obstacles perçus à la dialyse à domicile, les modalités favorisées (HD hospitalière contre HD à domicile), la distribution idéale des modalités au sein du programme local, la sensibilisation à l’HD à domicile et l’éducation des patients au sujet des modalités à domicile.

**Méthodes:**

Comparaisons par paires des réponses obtenues avant et à la suite de la formation.

**Résultats:**

Parmi les 115 membres du personnel infirmier en HD hospitalière, 100 se sont inscrits à la formation et 89 ont rempli à la fois les sondages qui précèdent et qui suivent la formation (taux de réponse de 89 %). Au départ, le personnel infirmier en HD hospitalière a ciblé un déficit cognitif, une faible force motrice et une faible acuité visuelle comme des obstacles à la dialyse péritonéale et à l’HD à domicile. L’HD hospitalière était favorisée pour la disponibilité des soins multidisciplinaires et du personnel médical en cas d’événement catastrophique. À la suite de la formation, l’opinion était généralement favorable à l’HD à domicile sans égard aux caractéristiques des patients, et à la majorité des facteurs patient/système. L’HD à domicile a été perçue comme étant sous-utilisée, tant au départ qu'à la suite de la formation. Finalement, à la suite de la séance de formation, le personnel infirmier en HD hospitalière était plus sensibilisé à l’HD à domicile, se sentait mieux informé de ses avantages, et plus à l’aise d’informer les patients en HD hospitalière quant aux modalités à domicile.

**Limites de l’étude:**

L’étude ne touche qu’un centre.

**Conclusions:**

Les initiatives d’éducation en soins infirmiers peuvent modifier l’opinion du personnel infirmier en HD hospitalière au sujet des modalités de dialyse à domicile, et devraient constituer un complément à la multitude de stratégies visant à promouvoir l’HD à domicile.

## What was known before

We previously identified that in-center HD nurses were more in favor of in-center HD compared with home dialysis for patients with certain characteristics and for some patient and system factors.

## What this study adds

We surveyed in-center HD nurses from another hospital institution before and after an education initiative aimed at home dialysis promotion. We found that perceptions were more favorable towards home dialysis after the initiative. This suggests that continuing nursing education initiatives are valuable at changing in-center HD nurses’ opinions towards home dialysis.

## Background

Nephrology health professionals are in favor of home dialysis (both peritoneal dialysis and home hemodialysis) due to the potential benefits to patients and providers; including improved survival, quality of life and lower treatment costs [1-9]. While in-center hemodialysis (HD) is still the most common form of dialysis therapy worldwide [10-12], it has been shown that nephrology nurses, physicians and nephrology administrators perceive that home dialysis is underutilized [13-17]. The discrepancy between opinion and reality may be due to a lack of pre-dialysis home modality education and the impression (by providers) that there are patient characteristics that are contraindications to home modalities [18-21].

Previous studies have suggested that nephrology nurses have positive views towards home modalities [22]. However, more recently, we have identified that in-center HD nurses prefer in-center HD for a number of patient and system factors [18]. There are multiple potential reasons behind this preference including a limited understanding of the benefits of home dialysis, an incomplete awareness of criteria for patient suitability for home modalities, and more frequent exposure of in-center HD nurses to home dialysis “failures” that require in-center HD [18,23].

Continuing nursing education (CNE) initiatives are common and have been found to be effective at enacting positive changes in nurses’ knowledge, attitudes and perceptions in a variety of areas of healthcare [24-28]. In-center HD nurses perceive that home dialysis CNE is valuable [18], but to our knowledge no previous study has identified whether CNE can alter opinions. Demonstrating a positive change in perceptions towards home dialysis would highlight the value of CNE initiatives in home dialysis promotion. Therefore, the purpose of this survey study was to determine if a CNE initiative delivered to in-center HD nurses could modify perceptions towards home dialysis. We hypothesized that perceptions would be more favorable towards home dialysis after the initiative.

## Methods

### Design and population

We conducted a cross-sectional survey study of all in-center HD nurses (those that worked in a HD facility) affiliated with a large Canadian quaternary care institute that attended a CNE initiative aimed at home dialysis promotion. The survey was conducted in May of 2013. In-center HD nurses included those that were “local” (working in one of two units with nephrologists on-site), and “satellite” (working in one of seven remote units with nephrologists accessible via telemedicine.) We included nurses working in remote satellite units as they make up a large part of the dialysis program. Nurses working in the home dialysis unit (caring for patients receiving home HD or peritoneal dialysis) or nephrology clinic were excluded.

### Administration of the survey

The survey was administered in paper format to all nurses 15 minutes preceding the CNE initiative, and repeated immediately following the initiative. Surveys were in sealed envelopes at the presentation site, and nurses located at the satellite facilities had surveys pre-delivered (in sealed envelopes) to each facility prior to the CNE. Survey responses were anonymous.

### Survey development

The survey was created using the template from our previous study conducted along the same theme [18]. This template was revised by a local expert panel using a modified Delphi process for content validity and to ensure optimal fit with the local context of patients receiving dialysis at our center. The expert panel (n = 11) consisted of nurse educators, home and in-center dialysis unit managers, a registered nurse with experience in quality improvement and a physician with expertise in home dialysis. Similar to the previous survey, identified domains included “nurse perceived barriers to home dialysis”, “home dialysis benefits”, “ability/knowledge/skill to encourage home modalities”, and “perceived ideal modality mix”. Potential survey questions were added and removed from each domain over two rounds of emails (each round conducted over two weeks) between expert panel members with allowance for open discussion around each potential question. A preliminary version of the survey was developed and distributed to all panel members to assess face validity and to make modifications to questions to improve understanding. All panel members agreed to the final version of the survey to be distributed. A summary of the key domains, questions and response options is noted in Table 1. The full version of the survey is available in Additional file 1: Figure S1.

**Table 1:** **Survey domains, questions and response items**

**Survey domain**	**Select questions**	**Select response options**
Nurse perceived barriers to home dialysis	-(1) Peritoneal dialysis and (2) Home hemodialysis can be performed on patients with:*	-No education after high school
		-Limited home space
		-Age greater than 70 years
		-Large body mass
		-Impaired cognition
		-Poor visual acuity
		-Poor motor strength
Home dialysis benefits	-Do you feel home dialysis (HD or PD) or in-center hemodialysis is more preferable for the following:**	-Reduced cost to patients
		-Reduced healthcare costs
		-Better patient survival
		-Better patient quality of life
Ability/knowledge/skill to encourage home modalities	- I am aware of home dialysis* modalities	Not applicable
	-I promote home dialysis to in-center HD patients*	
	-I am comfortable explaining home dialysis to patients*	
	-Promoting home dialysis will reduce employment for in-center HD nurses*	
Perceived ideal modality mix	-Given the current modality mix, in your opinion, what is the ideal proportion of patients that should receive each modality to maximize survival, wellness and quality of life?***	Not applicable

*5-item Likert; Strongly Agree; Agree; Neutral; Disagree; Strongly disagree.

**5-item Likert; In-Center Hemodialysis Strongly Preferred; In-Center Hemodialysis Somewhat Preferred; Neither Preferred; Home Dialysis Somewhat Preferred; Home Dialysis Strongly Preferred.

***Provided current modality mix: In-center hemodialysis 54%; Peritoneal dialysis 13%; Home hemodialysis 5%; Self-care hemodialysis 1%; Satellite unit hemodialysis 27%.

### The CNE initiative

The education session lasted three hours, and was offered at five different time points over one month to accommodate dialysis unit needs and staffing availability. Two of the five sessions used telehealth to provide more convenient access and facilitate greater participation from remote satellite units. Telehealth was offered to all seven satellite units, and six of the seven units attended through this medium.

The education session was delivered as a presentation and included the following:

1. Context of chronic kidney disease (CKD) in Nova Scotia (NS) and Canada including the current provincial transplant rate and home therapy rate
2. An examination of provincial home dialysis targets
3. A discussion on perceived benefits and advantages of home therapies
4. A discussion on perceived and actual barriers to home therapies and strategies to overcome these barriers (with some overlap with the Match-D tool [29]), including use of home care assistance for those with care dependence, poor vision and cognitive dysfunction [30,31]
5. The types of home therapies offered in the local renal program
6. Criteria for suitability of home dialysis
7. Training schedules for different home dialysis modalities
8. Cost comparisons between in-center HD, home peritoneal dialysis (PD) and home HD [6]
9. A review of educational resources available to staff

After the presentation, HD nurses viewed a twenty-two minute video on home dialysis that was developed by the Capital District Health Authority Renal Program to support the existing pre-dialysis patient modality education. The video included interviews with home HD, PD and self-care dialysis patients (patients who perform some or all of their dialysis treatment in a dialysis unit with limited supervision). In the video, patients discussed the rationale for their chosen modality, some of the challenges they overcame and the reasons why their chosen modality met their lifestyle needs. There were also short interviews with nursing staff from the pre-dialysis renal clinic as well as the home dialysis unit. The video is available online at http://vimeo.com/62701482.

### Analysis

Baseline demographics including age range, sex, years of dialysis nursing experience and proportion with the Canadian Nursing Association certification in nephrology nursing (CNeph(C)) were collected from the survey. The CNeph(C) involves a written examination and includes dialysis modality selection as a core element [32,33]. Demographics and nurse characteristics were described using univariate statistics. Likert Scale responses before and after the initiative were graphically displayed using proportions within each category and paired comparisons were made using the Wilcoxon signed-rank test. Comparisons of nurses’ opinions of ideal modality distribution before and after the education session were described with medians and interquartile ranges. Differences between ideal proportions before and after the initiative were also compared using the Wilcoxon signed-rank test. Statistical analyses were performed using Stata IC version 12 (StataCorp, College Station, TX), and a P value <0.05 was considered statistically significant. Institutional research ethics approval was obtained prior to conducting this study (Capital Health Research Ethics Board, CDHA-RS 2014-003).

## Results

### Characteristics of responders and response rate

100 out of a possible 115 nurses registered for the education sessions. A total of 89 attendees (77% of in-center HD nurses) completed pre and post surveys (response rate of 89%). Of the 89 respondents, 87 (98%) were female, 56 (63%) were between 31–50 years of age and 66 (74%) were in the first 10 years of nephrology nursing practice. The majority were in-center HD nurses (53, 60%). 5% of nurses had CNeph(C) certification. Baseline characteristics are noted in Table 2.

**Table 2:** **Demographic characteristics of the survey responders (N = 89)**

**Variable**	**n (%)**
Age range in years	
<31	16 (18)
31-40	26 (29)
41-50	30 (34)
51-60	13 (15)
>60	4 (4)
Female gender	87 (98)
Years of nephrology nursing	
<1	9 (10)
1-5	34 (38)
6-10	23 (26)
11-15	11 (12)
16-20	6 (7)
>20	6 (7)
In-center HD unit	
Local	53 (60)
Satellite	36 (40)
Nursing education in Canada	89 (100)
CNeph(C) certification*	4 (5)

*One missing response for CNeph(C) certification.

### Nurses’ perceptions of barriers to PD and HHD

Before the initiative, 27%, 29% and 29% of in-center HD nurses “strongly agreed” or “agreed” that PD could be performed for patients with impaired cognition, poor visual acuity, and poor motor strength. Similar responses were noted for home HD (19%, 20% and 26% for characteristics of impaired cognition, poor visual acuity and poor motor strength, respectively). Local and satellite unit nurses’ responses were similar for the majority of patient characteristics (data not shown). After the education initiative, perceptions were more positive towards PD and HHD for all characteristics studied, (Table 3, P < 0.001 for each characteristic).

**Table 3:** **Proportion of nurses selecting “agree” or “strongly agree” in response to the question:** ***“peritoneal dialysis or home hemodialysis can be performed on patients with the following characteristics”***

	Peritoneal dialysis*		Home hemodialysis*	
**Characteristic**	**Before initiative**	**After initiative**	**Before initiative**	**After initiative**
Poor socioeconomic status	80%	92%	73%	90%
Non-compliant with in-center HD	37%	90%	25%	84%
No education after high school	93%	97%	88%	98%
Limited home space	54%	85%	38%	69%
Multiple chronic illnesses	54%	93%	62%	91%
Age >70 years	74%	96%	72%	96%
No family caregivers	57%	93%	43%	85%
Large body mass	53%	96%	72%	97%
Impaired cognition	27%	81%	19%	79%
Poor visual acuity	29%	89%	20%	78%
Poor motor strength	29%	78%	26%	74%

*P < 0.001 for comparisons of each characteristic before and after initiative.

### Preference for home versus in-center HD

The baseline survey revealed that in-center HD was somewhat or strongly preferred for availability of multidisciplinary care (in 64% of responses) and presence of medical personnel in case of catastrophic events (in 46% of responses). These proportions fell to 30% and 25% after the initiative (P < 0.001). For quality of life, healthcare system cost, patient cost and patient survival, in-center HD was somewhat or strongly preferred in only 2%, 6%, 9% and 19% of responses. Preferences were similar comparing local and satellite unit responses. After the CNE initiative, nurses were more in favor of home dialysis for most patient and system factors (P < 0.01). Home dialysis was preferred for patient quality of life to a similar extent before and after the initiative (P = 0.06).

### Perceived ideal modality distribution

In-center HD nurses were asked what they believed the ideal modality distribution should be in the local renal program, given the current proportions (Table 4). The perspective was that PD and HHD should increase and that a smaller proportion should receive in-center HD or satellite HD. After the education initiative an even higher proportion of nurses felt that PD and HHD should increase compared to baseline. However, the magnitude of difference comparing perspectives before and after the intervention was small (Table 4).

**Table 4:** **Perceived ideal proportion of patients to receive each dialysis modality before and after education intervention**

**Modality (actual percentage at this center)**	**Perceived ideal distribution before intervention [median %, (Q1 to Q3)]**	**Perceived ideal distribution after intervention [median %, (Q1 to Q3)]**	**Difference in perceived ideal distribution (after versus before) [median %, (Q1 to Q3)]**	**P**
**In-center HD: local** (actual 54%)	26 (20 to 40)	25 (15 to 35)	0 (−7.5 to 0)	0.014
**HHD** (actual 5%)	20 (10 to 20)	20 (10 to 25)	0 (0 to 5)	0.029
**Self-Care** (actual 1%)	10 (5 to 12)	10 (5 to 15)	0 (−3 to 5)	0.123
**PD** (actual 13%)	20 (15 to 20)	20 (20 to 30)	0 (0 to 10)	0.0001
**In-center HD: satellite** (actual 27%)	20 (15 to 30)	20 (10 to 27)	0 (−10 to 0)	<0.0001

### Perception of home dialysis promotion and education

While baseline attitudes towards home dialysis were positive (with respect to awareness, perceived benefit, promotion of home dialysis and comfort with explaining home dialysis to patients), there were statistically significant improvements in most areas after the CNE initiative (P < 0.001). Only 18% and 16% of in-center HD nurses felt that promotion of home dialysis would reduce their employment before and after the initiative, respectively.

## Discussion

In this study, we found that the baseline perceptions of in-center HD nurses were favorable towards home dialysis, as was perceived ideal modality distribution. There were patient characteristics that were felt to be barriers to home dialysis including poor visual acuity, impaired cognition and poor motor strength. However, preferences shifted more positively towards home dialysis after the CNE session. Small increases were noted in awareness of home dialysis, perception of benefit and empowerment to educate patients after the CNE initiative.

This study emphasizes that CNE initiatives may be effective at changing opinions of healthcare workers to being more in-favor of home modality selection. To our knowledge, this is the first analysis of the direct effect of a CNE initiative on perceptions towards home dialysis. The potential impact of this in home dialysis is evident. It identifies that education of staff to emphasize and promote a “home first” attitude [16,34] is not a futile endeavor, and can be successfully delivered to a large group of nurses who spend a considerable amount of time interacting with in-center HD patients. Acknowledging that multiple small interventions may be needed to increase uptake of home dialysis [35], testing the effect of staff CNE on patient transfer to home dialysis after starting in-center HD would be a consideration for future study.

An important finding was that baseline perceptions of in-center HD nurses towards home dialysis were more positive than our previous survey study [18]. Interestingly, the proportion of patients on a home dialysis modality was higher at the center in which that previous survey was conducted. We speculate that the physical location of the in-center HD unit in relation to the home dialysis unit may influence perceptions of in-center HD nurses. In the previous study, home HD and PD operated independently, with different staff and in different locations. These locations were also separate from the in-center unit. In contrast, the home dialysis unit at this center is integrated to support both PD and home HD, and the in-center HD unit is located on the same floor in the same building. This physical proximity may increase awareness of home modalities and of patients receiving home dialysis at this center. Additionally, the home dialysis unit provides initial training to satellite HD nurses, so it is possible that this early exposure to home dialysis may influence their perceptions. In contrast, separation between the home dialysis units may limit awareness of either modality and potentially promote competition.

Although baseline perceptions were generally in favor of home dialysis, in-center HD nurses did not feel well informed about the benefits of home therapies or comfortable in explaining home dialysis to patients prior to the intervention. In-center HD nurses are not necessarily expected to advocate or promote home dialysis, however a lack of awareness of home dialysis or lack of confidence in the amount of knowledge or ability to convey attitudes about home dialysis may prevent some in-center HD nurses from taking a more active role in home dialysis promotion [23]. Facilitating this through CNE initiatives aimed at home dialysis promotion appears to be effective.

This study has a number of strengths. It builds on the previous survey study examining nurse’s opinions towards home dialysis, and emphasizes the need for a large-scale survey of multiple dialysis units to determine regional, national and international differences. The survey has both face validity and content validity. We were able to achieve a high rate of nurse participation in the CNE initiative, and a high survey response rate, which limits non-response bias.

There are limitations to this study. We only conducted the intervention and survey in a single center. It is possible that baseline perceptions and responses to education interventions might vary in other centers, where practice-patterns differ. However, the initiative was relatively simple and reproducible. There is potential difficulty in interpretability of responses to subjective questions. However, by using paired analyses, we ensured that changes in responses were collected within individual responders. Furthermore, given the short time frame between pre/post surveys, it is reasonable to assume that the criteria each responder used to define a subjective question would not be expected to change before and after the initiative. While our response rate was good, the group of nurses that did not participate in the CNE may have perceptions that are not favorable to home dialysis regardless of education. If they had participated, it might have reduced the impact of the CNE initiative. Finally while this study demonstrates a change in perception more favorably towards home dialysis, more research is needed to determine if this change improves the quality of home dialysis modality education delivered by in-center HD nurses (changed behavior) or uptake of home dialysis modalities for eligible patients (the intended observable outcome) [36]. Future studies examining the quality of informal education provided by in-center HD nurses and/or determining if CNE initiatives lead to an increase in home dialysis would be valuable.

## Conclusions

In this study of in-center HD nurses, we identified that baseline perceptions were favorable towards home dialysis for most patient characteristics and patient/system factors. A CNE initiative was effective at modifying opinions of in-center HD nurses towards home dialysis, and made in-center HD nurses more informed and more comfortable explaining home dialysis to patients. While an initiative such as this one can impact in-center HD nurses, it is not known if this increased knowledge and changed opinions will increase the uptake of home dialysis. We acknowledge that for home dialysis incidence and prevalence to increase, there needs to be a coordinated multi-pronged approach.

## Additional file

Additional file 1:
**Complete Survey.**

## Abbreviations

HD: Hemodialysis
IC/SAT HD: In-center and satellite hemodialysis
CNE: Continuing nursing education
CKD: Chronic kidney disease
NS: Nova Scotia
PD: Peritoneal dialysis
CNeph(C): Canadian Nursing Association certification in nephrology nursing
