# Supplementary material for: An education initiative modifies opinions of hemodialysis nurses towards home dialysis
Source: Can J Kidney Health Dis. 2015 Apr 28;2:16. doi: 10.1186/s40697-015-0051-z (PMC4411822; doi:10.1186/s40697-015-0051-z)
Supplement: Additional file 1: — Complete Survey. [file 40697_2015_51_MOESM1_ESM.docx]

|  | \| 1. \| Which dialysis unit do you currently spend the most time working in? \| \| \| \| --- \| --- \| --- \| --- \| \|  \| \|  \| In-Centre Hemodialysis Halifax \| \| --- \| --- \| \|  \| In-Centre Hemodialysis Dartmouth \| \|  \| Satellite Hemodialysis \| \| \| \| \| 2. \| Please indicate your gender. \| \|  \| \|  \| Female \| \| --- \| --- \| \|  \| Male \| \|  \|  \| \| \| 3. \| Please indicate your current age. \| \| \|  \| \|  \| Younger than 31 \| \| --- \| --- \| \|  \| 31-40 \| \|  \| 41-50 \| \|  \| 51-60 \| \|  \| Older than 60 \| \| \| \|  \|  \| \|  \|  \| \| \| --- \| --- \| \| 4. \| How many years have you been working as a hemodialysis nurse? \| \| \|  \| \|  \| Less than 1 year \| \| --- \| --- \| \|  \| 1-5 Years \| \|  \| 6-10 Years \| \|  \| 11-15 Years \| \|  \| 16-20 Years \| \|  \| More than 20 Years \| \|  \|  \| \| \| \| 5. \| Where did you initially learn nephrology nursing? \| \| \|  \| \|  \| Canada \| \| --- \| --- \| \|  \| United States \| \|  \| Europe \|   Other, please specify: \| \| \|  \|  \| \|  \|  \| \|  \| \| --- \| --- \| --- \| \| 6. \| Do you currently have your CNA Certification in nephrology nursing (C Neph(C))? \| \| \|  \| \|  \| Yes \| \| --- \| --- \| \|  \| No \| \| \| \|  \|  \| \| |
| --- | --- | --- | --- | --- | --- | --- | --- | --- | --- | --- | --- | --- | --- | --- | --- | --- | --- | --- | --- | --- | --- | --- | --- | --- | --- | --- | --- | --- | --- | --- | --- | --- | --- | --- | --- | --- | --- | --- | --- | --- | --- | --- | --- | --- | --- | --- | --- | --- | --- | --- | --- | --- | --- | --- | --- | --- | --- | --- | --- | --- | --- | --- | --- | --- | --- | --- | --- | --- | --- | --- | --- | --- | --- | --- | --- | --- | --- | --- | --- | --- | --- | --- | --- | --- | --- | --- | --- | --- | --- | --- | --- | --- | --- | --- | --- | --- | --- |

|  | \| 7. \| Once a patient begins in-centre or satellite hemodialysis, who do you believe has the MOST influence on their choice to remain on in-centre or satellite hemodialysis? Please rank each option from MOST (1) to LEAST (4) \| \| --- \| --- \| \|  \| \|  \| 1 \| 2 \| 3 \| 4 \| \| --- \| --- \| --- \| --- \| --- \| \| Nephrologist or nurse practitioner \|  \|  \|  \|  \| \| In-centre or satellite hemodialysis nurse \|  \|  \|  \|  \| \| Patient him/herself \|  \|  \|  \|  \| \| Family member or caregiver \|  \|  \|  \|  \| \| \|  \|  \|  \| 8. \| Please rate how much you agree or disagree with the following statements:  **Peritoneal dialysis** can be performed on patients with: \| \| --- \| --- \| \|  \| \|  \| **Strongly agree** \| **Agree** \| **Neutral** \| **Disagree** \| **Strongly Disagree** \| \| --- \| --- \| --- \| --- \| --- \| --- \| \| Poor socio-economic status \|  \|  \|  \|  \|  \| \| No education after high school \|  \|  \|  \|  \|  \| \| Non-compliance with in-centre hemodialysis \|  \|  \|  \|  \|  \| \| Limited home space \|  \|  \|  \|  \|  \| \| Multiple chronic illnesses \|  \|  \|  \|  \|  \| \| Age greater than 70 years \|  \|  \|  \|  \|  \| \| No family caregivers \|  \|  \|  \|  \|  \| \| Large body mass \|  \|  \|  \|  \|  \| \| Impaired cognition \|  \|  \|  \|  \|  \| \| Poor visual acuity \|  \|  \|  \|  \|  \| \| Poor motor strength \|  \|  \|  \|  \|  \| \| |
| --- | --- | --- | --- | --- | --- | --- | --- | --- | --- | --- | --- | --- | --- | --- | --- | --- | --- | --- | --- | --- | --- | --- | --- | --- | --- | --- | --- | --- | --- | --- | --- | --- | --- | --- | --- | --- | --- | --- | --- | --- | --- | --- | --- | --- | --- | --- | --- | --- | --- | --- | --- | --- | --- | --- | --- | --- | --- | --- | --- | --- | --- | --- | --- | --- | --- | --- | --- | --- | --- | --- | --- | --- | --- | --- | --- | --- | --- | --- | --- | --- | --- | --- | --- | --- | --- | --- | --- | --- | --- | --- | --- | --- | --- | --- | --- | --- | --- | --- | --- | --- | --- | --- | --- | --- | --- | --- | --- | --- |

| 9. | Please rate how much you agree or disagree with the following statements:  **Home Hemodialysis** can be performed on patients with: |
| --- | --- |
|  | \|  \| **Strongly agree** \| **Agree** \| **Neutral** \| **Disagree** \| **Strongly Disagree** \| \| --- \| --- \| --- \| --- \| --- \| --- \| \| Poor socio-economic status \|  \|  \|  \|  \|  \| \| No education after high school \|  \|  \|  \|  \|  \| \| Non-compliance with in-centre hemodialysis \|  \|  \|  \|  \|  \| \| Limited home space \|  \|  \|  \|  \|  \| \| Multiple chronic illnesses \|  \|  \|  \|  \|  \| \| Age greater than 70 years \|  \|  \|  \|  \|  \| \| No family caregivers \|  \|  \|  \|  \|  \| \| Large body mass \|  \|  \|  \|  \|  \| \| Impaired cognition \|  \|  \|  \|  \|  \| \| Poor visual acuity \|  \|  \|  \|  \|  \| \| Poor motor strength \|  \|  \|  \|  \|  \| |

| 10. | Do you feel home dialysis (HD or PD) or in-centre hemodialysis is more preferable for the following reasons: | | |
| --- | --- | --- | --- |
| \|  \| **In-centre hemodialysis strongly preferred** \| **In-centre hemodialysis somewhat preferred** \| **Neither in-centre or home dialysis preferred** \| **Home dialysis somewhat preferred** \| **Home dialysis strongly preferred** \| \| --- \| --- \| --- \| --- \| --- \| --- \| \| Reduced cost to patients (including lost employment time) \|  \|  \|  \|  \|  \| \| Reduced cost to healthcare system \|  \|  \|  \|  \|  \| \| Lower risk of catastrophic events to patients \|  \|  \|  \|  \|  \| \| Better patient survival \|  \|  \|  \|  \|  \| \| Better patient quality of life \|  \|  \|  \|  \|  \| \| Availability of multidisciplinary and physician support \|  \|  \|  \|  \|  \| | | |  |
| 11. | The current proportion of dialysis patients receiving each modality at CDHA is shown in brackets.  In your opinion, what is the IDEAL proportion of patients that should receive each modality to maximize survival, wellness, and quality of life? The total should add up to 100.   \|  \| **Ideal % per Modality** \| \| --- \| --- \| \| In-centre (Halifax or Dartmouth) hemodialysis (54%) \|  \| \| Peritoneal dialysis (13%) \|  \| \| Home hemodialysis (5%) \|  \| \| Self-care hemodialysis (1%) \|  \| \| Satellite hemodialysis (27%) \|  \| \| **TOTAL** \| **100%** \| | | |
|  | |  |  |

| 12. | For each of the following statements, place a check mark in the box that best describes your answer. | |
| --- | --- | --- |
| \|  \| **Strongly Agree** \| **Agree** \| **Neutral** \| **Disagree** \| **Strongly Disagree** \| \| --- \| --- \| --- \| --- \| --- \| --- \| \| I am aware of home dialysis modalities \|  \|  \|  \|  \|  \| \| I believe home dialysis is beneficial \|  \|  \|  \|  \|  \| \| I promote home dialysis to in-centre/satellite hemodialysis patients \|  \|  \|  \|  \|  \| \| I am well informed about the benefits of home dialysis \|  \|  \|  \|  \|  \| \| I am comfortable explaining home dialysis to my patients \|  \|  \|  \|  \|  \| \| If I knew more about home dialysis I would promote it to my patients \|  \|  \|  \|  \|  \| \| Promoting home dialysis will reduce employment for in-centre/satellite hemodialysis nurses \|  \|  \|  \|  \|  \| | |  |
